# G388R mutation of the *FGFR4* gene is not relevant to breast cancer prognosis

**DOI:** 10.1038/sj.bjc.6601450

**Published:** 2004-01-06

**Authors:** P Jézéquel, L Campion, M-P Joalland, M Millour, F Dravet, J-M Classe, V Delecroix, R Deporte, P Fumoleau, G Ricolleau

**Affiliations:** 1 Département de Biologie Oncologique, Centre Régional de Lutte Contre le Cancer, René Gauducheau, boulevard Jacques Monod, 44805 Saint Herblain, France; 2 Unité de Biostatistique, Centre Régional de Lutte Contre le Cancer, René Gauducheau, boulevard Jacques Monod, 44805 Saint Herblain, France; 3 Service d'Oncologie Médicale, Centre Régional de Lutte Contre le Cancer, René Gauducheau, boulevard Jacques Monod, 44805 Saint Herblain, France; 4 Service de Chirurgie Oncologique, Centre Régional de Lutte Contre le Cancer, René Gauducheau, boulevard Jacques Monod, 44805 Saint Herblain, France

**Keywords:** breast cancer, FGFR4 genotype, G388R mutation, prognostic marker, tyrosine kinase

## Abstract

This study screened large cohorts of node-positive and node-negative breast cancer patients to determine whether the G388R mutation of the *FGFR4* gene is a useful prognostic marker for breast cancer as reported by Bange *et al* in 2002. Node-positive (*n*=139) and node-negative (*n*=95) breast cancer cohorts selected for mutation screening were followed up for median periods of 89 and 87 months, respectively. PCR – RFLP analysis was modified to facilitate molecular screening. Curves for disease-free survival were plotted according to the Kaplan – Meier method, and a log-rank test was used for comparisons between groups. Three other nonparametric linear rank-tests particularly suitable for investigating possible relations between G388R mutation and early cancer progression were also used. Kaplan – Meier analysis based on any of the four nonparametric linear rank tests performed for node-positive and node-negative patients was not indicative of disease-free survival time. G388R mutation of the *FGFR4* gene is not relevant for breast cancer prognosis.

Many components of mitogenic signalling pathways have been identified over the past 15 years, including the large family of protein kinases. Dysregulation of these biological processes may play an important role in the growth and survival of cancer cells. The 20 members of the fibroblast growth factor (FGF) family transduce a variety of biological signals via distinct transmembrane tyrosine kinase receptors (FGFR1 – FGFR4) encoded by four closely related genes (Powers *et al*, 2000). Mature FGFR proteins, which act as membrane-spanning tyrosine kinase receptors, have an extracellular ligand-binding domain, a transmembrane domain and a split intracellular tyrosine kinase domain (Green *et al*, 1996). Fibroblast growth factor -mediated signals have mitogenic, angiogenic and hormone regulatory effects and play key roles in a wide variety of crucial biological activities requiring cell growth, differentiation, migration and chemotaxis. Molecular anomalies of *FGFR* genes (inappropriate expression, single nucleotide polymorphism (SNP), splice variations, genomic alterations) have been described in several types of human cancer (bladder, cervical, colorectal carcinomas and multiple myeloma) and in skeletal deformities (achondroplasia, Crouzon syndrome and thanatophoric dysplasia type II) (Muenke and Schell, 1995; Avet-Loiseau *et al*, 1998; Cappellen *et al*, 1999; Vajo *et al*, 2000. Bange *et al*, (2002) recently studied the role of an SNP responsible for a missense mutation (G388R) in the transmembrane domain of FGFR4, in the progression and prognosis of nodepositive breast cancer. This G → A transition changes the sense of codon 388 from Gly (GGG) to Arg (AGG). The authors concluded that the G388R mutation in heterozygous or homozygous state was significantly over-represented in node-positive breast cancer patients with early relapse, but has no role in tumour formation, making this SNP a prognostic marker. On a worldwide basis, they found that allele distribution in normal controls and breast cancer patients did not differ significantly, showing an estimated 45.4% for the Gly/Gly allele, 45.7% for the Gly/Arg allele, and 8.9% for the Arg/Arg allele (Bange *et al*, 2002). No relationship has been found between this SNP and the prognosis for node-negative breast cancer.

Our aim was to confirm the findings of Bange *et al* on a larger cohort, using an improved PCR – RFLP analysis and a reinforced and more adapted statistical analysis for investigating the possible association between G388R mutation and early cancer progression. Screening was conducted in a cohort of node-positive breast cancer patients who received different adjuvant therapies (endocrine (*n*=67), or chemotherapeutic (*n*=72)) and in a cohort of node-negative breast cancer patients (*n*=95). These cohorts were followed up, respectively, for median periods of 89 and 87 months. PCR – RFLP analysis was modified to make molecular screening more convenient and less time-consuming. Curves for disease-free survival (DFS) were plotted according to the Kaplan – Meier method, and the log-rank test was used for comparisons between groups, as in the study of Bange *et al*, (Kaplan and Meier, 1958; Cox and Oakes, 1984). Statistical analysis was reinforced by using three other nonparametric linear rank tests (Breslow, Peto – Prentice and Tarone – Ware) (Breslow, 1970; Peto and Peto, 1972; Tarone and Ware, 1977; Prentice, 1978).

## MATERIALS AND METHODS

### Patients

The study included 234 consecutive unselected women with primary breast tumours, who were diagnosed and treated primarily between 1988 and 1997 at the René Gauducheau Cancer Center. Informed consent was obtained from patients to use their surgical specimens and clinicopathological data for research purposes, as required by the French Committee for the Protection of Human Subjects. These patients showed no evidence of distant metastasis at the time of diagnosis. None had received chemotherapy, endocrine therapy or radiation therapy prior to surgery. Treatment decisions were based solely on consensus recommendations at the time of diagnosis. Patients were followed up for DFS (delimited by the first clinically recognised evidence of local or distant recurrence). Node-positive cohort was composed of 139 patients (mean age 55.4 years, range 32–80), who received different adjuvant therapies (tamoxifen, *n*=67; 5-fluorouracil (500 mg/m^−2^), epirubicin (50 mg/m^−2^) and cyclophosphamide (500 mg/m^−2^) (FEC50), *n*=72) after primary surgery and postoperative radiation therapy. Node-positive patients were followed up every 4 months during 2 years, then every 6 months during 3 years, and annually thereafter. Clinical examination, mammography and chest radiography were performed twice a year, and bone scintigraphy and liver ultrasonography annually. The node-negative cohort was composed of 95 patients (mean age 57.1 years, range 34–78), all of whom were treated only by primary surgery and postoperative radiation therapy (median follow-up 87 months, range 58–164). The follow-up of node-negative patients included clinical examination, mammography and chest radiography every 6 months during 2 years and annually thereafter. The clinicopathologic characteristics of the patients are indicated in Table 1

**Table 1:** Clinicopathologic characteristics of the two cohorts

	**N+**	**N−**
No. of patients	139	95
Mean age at diagnosis (years)	55.4	57.1
Median follow-up (months)	89	87
Relapse	57	25
Primary surgery	139	95
Adjuvant radiation therapy	139	95
Adjuvant endocrine therapy (Tam)	67	0
Adjuvant chemotherapy (FEC50)	72	0
		
*Histological type*		
Infiltrating ductal carcinoma	121	70
Infiltrating lobular carcinoma	8	11
Others	10	14
		
*No. of positive axillary lymph nodes*		
0	0	95
1 – 3	109	0
>3	30	0
		
*Tumor size (mm)*		
< 20	27	51
⩾20	99	41
Not determined	13	3
		
*Elston Ellis histological grade*		
I	9	30
II	68	48
III	45	4
Not determined	17	13
		
*Hormone receptor status*		
ER+/PgR+	88	67
ER−/PgR+	13	3
ER+/PgR-	26	12
ER−/PgR-	19	12
Not determined	3	1
		
*Relapse site*		
Local only	14	11
Any distant site	43	14

N−= node-negative; N+= node-positive, Tam= tamoxifen; ER=oestrogen receptor, PgR= progesterone receptor.

.

### DNA samples

All tumour tissue samples were surgically collected, typed by a pathologist and grossly dissected before being snap-frozen in liquid nitrogen. To exclude the presence of ‘contaminating’ cancer-associated somatic mutations affecting the locus of interest or loss of heterozygosity, 70 randomly selected patients were screened both in tumour tissue and in peripheral white blood cells (WBCs). Peripheral venous blood samples were taken just before breast resection or mastectomy. DNA was isolated according to standard protocols from peripheral WBCs and from shock-frozen grossly dissected breast cancer tissues.

### PCR – RFLP analysis

The primers described by Bange *et al* were applied to screen for G388R mutation, but with the following modifications: PCR-mediated site-directed mutagenesis used the PCR forward primer with a single base mismatch (C → A: in bold type) to destroy a constant *Bst*NI restriction site that complicated visualisation of the restriction profile of the corresponding PCR product, and a guanine was added to the 3′ end of this primer to improve annealing (5′ GACCGCAGCAGCGCCCGAGGC**A**AG**G** 3′). In the reverse primer, an adenine was turned into a cytosine (in bold type), according to the GenBank sequence (accession number: Y13901) (5′ AGAGGGAAG**C**GGGAGAGCTTCTG 3′). Under these conditions, the undigested PCR product had a length of 168 bp. The G → A transition in codon 388 created a new *Bst*NI restriction site (CC ↓ WGG). As a positive control for the digestion efficiency of *Bst*NI, an additional *Bst*NI restriction site was located in the 168 bp PCR product.

Reactions were performed with 500 ng of genomic DNA in a total volume of 50 *μ*l containing (final concentration) 10 mM Tris-HCl (pH 8.3), 50 mM KCl, 2.5 mM MgCl_2_, 0.25 mM of the four deoxynucleotide triphosphates, 1 U of *Taq* polymerase (Applied Biosystems, Branchburg, NJ, USA) and 0.25 *μ*M of each primer. PCR conditions were as follows: preliminary denaturation at 95°C for 5 min, followed by 35 cycles of 30 s at 95°C and 45 s at 72°C. The reaction terminated with 5 min at 72°C. The digestion reactions contained 10 *μ*l of PCR product, 0.5 *μ*l of *Bst*NI (5 U; New England Biolabs, Beverly, MA, USA), 2 *μ*l of 10 × NEBuffer 2 (supplied with the enzyme) and 0.2 *μ*l bovine serum albumin (100 mg l^−1^) in a final volume of 20 *μ*l. These components were incubated for 60 min at 60°C. After the reaction ended, 10 *μ*l of the PCR mixture were mixed with a loading buffer and electrophoresed in a 4% agarose 1000® gel (Life Technologies, Carlsbad, CA, USA). Bands were visualised by ethidium bromide staining of the gel.

### Statistical methods

The variable of interest was DFS, and survival curves for DFS were plotted according to the Kaplan – Meier method. Comparison between groups was performed by the log-rank (Mantel – Cox) test, as in the study of Bange *et al*, and three other non-parametric linear rank tests were added (Breslow, Tarone – Ware and Peto – Prentice). These four tests used to assess the equality of the survivor function across groups are members of a family of statistical tests that serve as extensions to the censored data of traditional nonparametric rank tests for comparison of two or more distributions. Quite simply, the contribution to the statistical test is obtained at each distinct relapse time in the data as a weighted standardised sum of differences between the observed and expected number of events in each of the K groups. The expected number of events is obtained under the null hypothesis of no differences in the global survival experience of the K groups. The weight function used determines the statistical test: 1 for the log-rank test, *n*_*i*_ (number of subjects in the risk pool at each relapse time *i*) for the Breslow test, *n*_*i*^*1/2*^_ for the Tarone – Ware test, and *S*_(*ti*)_ (estimated Kaplan – Meier survivor fraction value at each relapse time *i*) for the Peto – Prentice test. The earlier the relapse time, the greater the weight. Thus, these tests are quite suited for investigating the possible relation between G388R mutation and early cancer progression. All tests were performed at a significance level of α=0.05. Two groups were followed, as in the study of Bange *et al*: Gly/Gly alleles and Gly/Arg alleles plus Arg/Arg alleles. Analyses were performed using the BMDP statistical software (Dixon, 1981).

## RESULTS

### PCR – RFLP analysis

**Figure 1:**
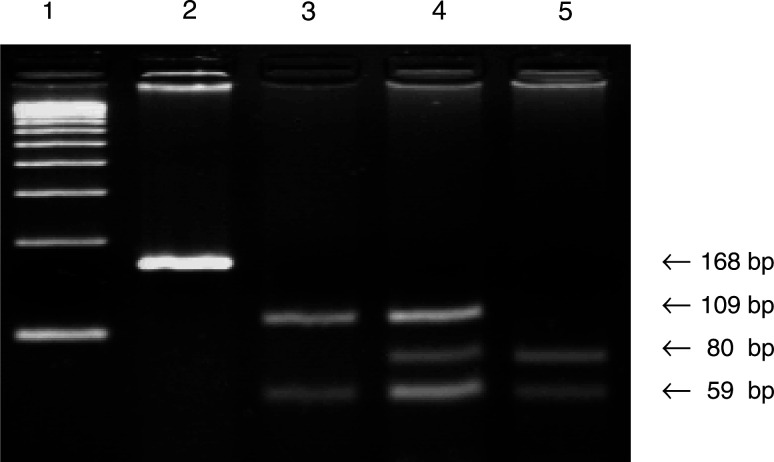
PCR – RFLP analysis of *FGFR4* gene G388R mutation. Lane 1, *Hae*III-digested pBR322 size marker; lane 2, amplification control; lane 3, wild-type control (Gly/Gly), *Bst*NI – digested; lane 4, heterozygote carrier (Gly/Arg), *Bst*NI – digested; lane 5, homozygote carrier (Arg/Arg), *Bst*NI – digested.

After digestion with *Bs*tNI, the wild-type allele produced two fragments of 109 and 59 bp. Conversely, the PCR product containing the G → A transition in heterozygous state produced four fragments of 109, 80, 59 and 29 bp. The homozygous state produced three fragments of 80, 59 and 29 bp. The 29-bp fragment was not visible on agarose gel (Figure 1

).

### Germinal mutation

The same genotype was found in 70 tumour tissues and corresponding peripheral WBCs, which proved the germinal origin of the G388R mutation.

### Allele distribution

Allele distribution of the G388R mutation in our local breast cancer population showed 51.7% of patients with homozygous Gly/Gly alleles, 37.2% with heterozygous Gly/Arg alleles, and 11.1% with homozygous Arg/Arg alleles. Allele distribution frequency between node-positive and node-negative patients was not significantly different (*χ*^2^=0.38; *P*=0.827; data not shown). The results of G388R mutation screening are summarised in Table 2

**Table 2:** *FGFR4* G388R allele distribution in the two breast cancer cohorts

		**Gly/Gly**		**Gly/Arg**		**Arg/Arg**	
	*n*	*n*	%	*n*	%	*n*	%
N+	139	73	52.5	52	37.4	14	10.1
N−	95	48	50.5	35	36.9	12	12.6

. Allele distribution frequency between our patients and the groups studied by Bange *et al* was not significantly different (*χ*^2^=4.3; *P*=0.12; data not shown). The G388R mutation appears to be as widespread in non-Caucasian (Chinese) as Caucasian populations (Bange *et al*, 2002).

### Usefulness of the FGFR4 genotype as a breast cancer prognostic marker

As shown in Table 3

**Table 3:** Association between *FGFR4* alleles and pathological parameters

	**N+**			**N−**		
	**Arg/Arg Gly/Arg**	**Gly/Gly**	***P***	**Arg/Arg Gly/Arg**	**Gly/Gly**	***P***
Age at diagnosis (years)	55.9 (12.5)^a^	54.9 (11.4)	0.64	56.1 (11.7)	58.1 (11.2)	0.41
Tumour size (mm)	27.6 (12.9)	30.7 (17.7)	0.27	17.6 (6.7)	19.2 (10.4)	0.38
Elston Ellis histological grade						
I	3	6		13	17	
II	34	34	0.64	25	23	0.75
III	22	23		2	2	
Hormone receptor status						
ER+/PgR+	43	45		33	34	
ER-/PgR+	4	6	0.93	3	0	0.34
ER+/PgR−	10	9		6	6	
ER-/PgR−	9	10		5	7	

N−=node-negative, N+= node-positive, ER= oestrogen receptor, PgR= progesteron receptor.

^a^Mean (s.d.).

, no correlation was observed between the FGFR4 allele and pathological parameters such as age at diagnosis, tumour size, Elston Ellis histological grade and hormone receptor status in node-postive and in node-negative patients.

**Figure 2:**
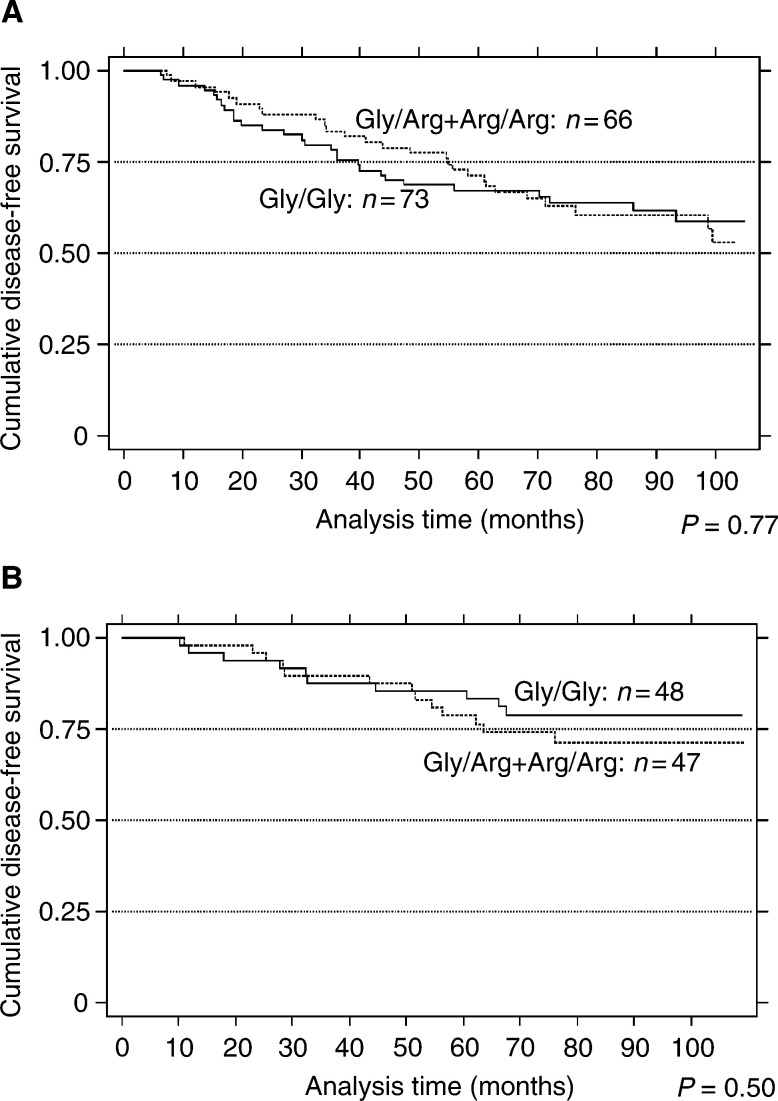
G388R mutation of the *FGFR4* gene is not related to increased tumours progression. Kaplan – Meier disease-free survival estimate in node-positive **(A)**, and node-negative **(B)** patients, according to *FGFR4* allele distribution. *P*-values were calculated by the Breslow test for comparisons between groups.

Kaplan – Meier survival analysis by any of the four nonparametric linear rank tests, when performed for node-positive patients, did not show any statistically significant difference in DFS according to G388R status, which is contrary to the findings of Bange *et al* (Figure 2

). The *P*-values of the different tests are indicated in Table 4

**Table 4:** *P*-values of the four nonparametric linear rank tests used

	**N+**	**N−**
Log-rank	0.83	0.44
Breslow	0.77	0.50
Tarone – Ware	0.92	0.47
Peto – Prentice	0.92	0.46

. Power calculation strengthened our negative results for node-positive patients (1−β=0.92) (Freedman, 1982). As Bange *et al* showed, no relation for survival was found between DFS time and G388R status in node-negative patients.

## DISCUSSION

In conclusion, the G388R mutation of the *FGFR4* gene does not appear to be an effective prognostic marker of breast cancer, contrary to the findings of Bange *et al*. Larger patient cohorts (139 *vs* 46 node-positive patients) and powerful statistical methods (Breslow, Tarone – Ware or Peto – Prenctice) were used in the present study, but no statistically significant difference in DFS according to G388R status was detected during early follow-up. Since complete clinicopathologic characteritics from Bange *et al* cohorts are not available, such as age at diagnosis, tumour size, histological grade, pN and hormone receptor status, we cannot exclude a possible link between one of these pathological parameters and the G388R mutation, which could create some confounding in the survival analysis.

Recent studies have focused on the possible implication of FGFR4 in the carcinogenesis of different cancers (Coope *et al*, 1997; Olson *et al*, 1998; Hart *et al*, 2000; Cavallaro *et al*, 2001; La Rosa *et al*, 2001; Ezzat *et al*, 2002; Shah *et al*, 2002). All these results indicate the complexity of the FGFR4 signalling pathway and the weak frontier separating normal from malignant cell proliferation. The role of FGFR4 in carcinogenesis is still under investigation, notably the relation between genotype and phenotype, but also its involvement in cancer progression via paracrine/autocrine modulation of FGFR4 ligands and FGFR4 and its possible implication in an oncogenic multistep process, as described for FGFR3 in multiple myeloma (Chesi *et al*, 2001). However, further studies are needed to explore the role of this mutation as a molecular marker and the possible value of the *FGFR4* gene or protein as a target for cancer therapy.
